# Activation of sensory cortex by imagined genital stimulation: an fMRI analysis

**DOI:** 10.3402/snp.v6.31481

**Published:** 2016-10-25

**Authors:** Nan J. Wise, Eleni Frangos, Barry R. Komisaruk

**Affiliations:** Department of Psychology, Rutgers University – Newark, Newark, NJ, USA

**Keywords:** human female, tactile imagery, clitoris, nipple, self-stimulation, homunculus, sexual arousal, genitalia, primary somatosensory cortex, secondary somatosensory cortex

## Abstract

**Background:**

During the course of a previous study, our laboratory made a serendipitous finding that just thinking about genital stimulation resulted in brain activations that overlapped with, and differed from, those generated by physical genital stimulation.

**Objective:**

This study extends our previous findings by further characterizing how the brain differentially processes physical ‘touch’ stimulation and ‘imagined’ stimulation.

**Design:**

Eleven healthy women (age range 29–74) participated in an fMRI study of the brain response to imagined or actual tactile stimulation of the nipple and clitoris. Two additional conditions – imagined dildo self-stimulation and imagined speculum stimulation – were included to characterize the effects of erotic versus non-erotic imagery.

**Results:**

Imagined and tactile self-stimulation of the nipple and clitoris each activated the paracentral lobule (the genital region of the primary sensory cortex) and the secondary somatosensory cortex. Imagined self-stimulation of the clitoris and nipple resulted in greater activation of the frontal pole and orbital frontal cortex compared to tactile self-stimulation of these two bodily regions. Tactile self-stimulation of the clitoris and nipple activated the cerebellum, primary somatosensory cortex (hand region), and premotor cortex more than the imagined stimulation of these body regions. Imagining dildo stimulation generated extensive brain activation in the genital sensory cortex, secondary somatosensory cortex, hippocampus, amygdala, insula, nucleus accumbens, and medial prefrontal cortex, whereas imagining speculum stimulation generated only minimal activation.

**Conclusion:**

The present findings provide evidence of the potency of imagined stimulation of the genitals and that the following brain regions may participate in erogenous experience: primary and secondary sensory cortices, sensory-motor integration areas, limbic structures, and components of the ‘reward system’. In addition, these results suggest a mechanism by which some individuals may be able to generate orgasm by imagery in the absence of physical stimulation.

We reported previously that in response to self-stimulation, the clitoris, vagina, cervix, and nipple activate differentiable regions of the paracentral lobule – that is, the genital region of the primary sensory cortex or ‘genital sensory cortex’ (Komisaruk, Wise, Frangos, Liu, et al., 2011). In the course of this study, a serendipitous finding arose from one of our control conditions, during which the participants were instructed to ‘think’ about self-stimulating their clitoris, vagina, and nipple; we observed that their just imagining stimulation of these regions generated activity in the genital sensory cortex that overlapped with that induced by actual tactile stimulation, although of a lesser magnitude. In addition, imagining the stimulation appeared to activate the frontal cortical regions substantially more than did the corresponding tactile stimulation (Wise, Frangos, & Komisaruk, 2010).

These findings are consistent with recent studies demonstrating that imagining stimulation of specific body regions activates corresponding regions of the primary and secondary somatosensory cortices (S1, S2) and the insula, although to a lesser degree than the actual tactile stimulation. By contrast, the magnitude of activation of the inferior parietal lobule, medial frontal gyrus, dorsolateral prefrontal areas, and inferior frontal gyrus is greater for the tactile imagery conditions (Olivetti Belardinelli et al., 2009; Yoo, Freeman, McCarthy, & Jolesz, 2003). Of particular interest is S2, as it is believed to participate in aspects of somatosensory attention (Chen et al., 2008), experimentally induced pain in women suffering from vulvar vestibulitis (Pukall et al., 2005), and in the interpretation of sensation as erotic (Georgiadis et al., 2006).

As pointed out by Cazala, Vienney, and Stoleru (2015), it is important that further studies be done to elucidate the mechanisms underlying the sexually stimulating and pleasurable qualities of genital sensations. This study is responsive to this by comparing the effect on brain activity of imagined stimulation by speculum versus dildo to systematically explore the differences between imagery that has a prosaic versus an erotic context. This study extends our serendipitous finding into a more extensive investigation of how the brain differentially processes physical ‘touch’ stimulation and mental ‘imagined’ stimulation of the nipple and clitoris.

## Methods

### Research participants

Eleven healthy right-handed women (age range 29–74, *M*=43.6, SD=13.6) were recruited for this study by word of mouth. One participant was post-menopausal; the other women were pre- or perimenopausal. Each participant gave informed consent as per the University Institutional Review Board for this approved study. Prior to the scanning procedure, they were interviewed about their sexual histories. All participants indicated that they were experienced with use of dildos, and all had experience with gynecological examinations via speculum.

The scanning session took place at the University Brain Imaging Center, in compliance with all MRI common practices. Participants were paid $50 for their participation in the study. Following the scanning procedure, all participants were interviewed about their experience in the scanner, including a request to rate their arousal, and the vividness and potential aversiveness of the imagery conditions.

### Experimental paradigm

After acquisition of localizers and magnetization prepared rapid acquisition gradient-echo (MPRAGE) anatomical images, the participants followed instructions presented visually on an fMRI-compatible screen.

The following is an overview of the protocol sequence: rest–imagined tactile stimulation of nipple–imagined tactile stimulation of the clitoris–tactile stimulation of the nipple–tactile stimulation of the clitoris–rest–imagine speculum–imagine dildo–imagine speculum. The modeling ‘control’ was intercalated with each of these conditions, with the exception of the imagine speculum–imagine dildo sequence.

The following is a detailed description of the protocol sequence:

For the first 60 s, the participants were instructed to rest. The experimental protocol consisted of four 5-min trials in the following order: nipple imagine stimulation (NIS), clitoris imagine stimulation (CIS), nipple touch stimulation (NTS), and clitoris touch stimulation (CTS). Each trial consisted of 30 s of control, ‘modeling’ of either the physical or imagined stimulation followed by 30 s of engaging in either mental imagery or physical stimulation ‘to comfortable intensity’ as instructed, repeating five times in succession for a total of 5 min. ‘Modeling’ consisted of making the hand movements to rhythmically stimulate the nipple without actually touching it. This alternated with 30 s of actual nipple touch, during which the participant was cued to use her right hand to rhythmically stimulate her left nipple. This sequence of nipple ‘model’ and nipple ‘touch’ alternated five times in succession for a total of 5 min. Comparable procedures were used for the clitoral touch and modeling condition. For the imagery trials, the ‘model’ condition was analogous to the model condition for the physical trials, but the participant was instructed to just think about making the modeling movements rather than actually executing them. For example, during the NIS trial, the participant was first instructed to ‘think model’, which cues her to think about making rhythmic movements with her left hand over her right nipple for 30 s. Then she saw the instruction, ‘think nipple stimulate’, which cued her to *imagine* rhythmically touching her left nipple with her right hand for 30 s. This sequence repeated five times for a total of 5 min. The CIS trial, likewise, alternated 30 s of imagined right hand ‘model’ movements with 30 s of imagined stimulation of the clitoris for a total of 5 min.

The protocol sequence began with the imagery trials to avoid the potential priming effects that actual tactile stimulation could induce. The tactile stimulation trials started with nipple rather than clitoris stimulation to avoid the potential confound of any lingering effects from stimulation of the clitoris. Because of these concerns, the conditions were not counterbalanced and the trials were always presented in the following order: NIS, CIS, NTS, and CTS.

Following completion of the imagery and physical stimulation trials, after a brief rest the experiment concluded with an additional imagery sequence during which the participant viewed instructions to ‘imagine speculum’ (to think about having a speculum inserted into her vagina by another person) for 30 s, followed by instructions to ‘imagine dildo’ (to think about having a dildo inserted into her vagina by another person) for 60 s, and ending with another trial of ‘imagine speculum’ for the final 30 s.

In a post-scan interview, the participants rated the vividness of their imagery experiences during the various imagery conditions on a scale of 1 (no image/sensation) to 7 (very vivid image/sensation). They were asked to indicate if any of the imagery conditions were aversive. They were also asked to rate how sexually aroused they were from 1 (low) to 7 (high) during each of the physical stimulation and imagined stimulation conditions.

### fMRI acquisition

The fMRI scans were performed using a 3T Siemens Trio with a Siemens 12-channel head coil. For registration purposes, anatomical images were acquired using MPRAGE sequences (176 slices in the sagittal plane using 1-mm-thick isotropic voxels, TR/TE=1900/2.52 ms, field of view=256, 256×256 matrix, flip angle=9 degrees; 50% distance factor). For the experimental scan, gradient-echo Echo-planar imaging (EPI) sequences were acquired of the whole brain including the entire medulla oblongata (33 slices in the axial plane using 3-mm isotropic voxels, TR/TE=2000/30 ms, interslice gap=1.5 mm, flip angle=90, field of view=192, 64×64).

### Head immobilization assembly

Head movement during the experimental tasks was minimized (mean displacement=0.4 mm) through the use of a combination of two different types of commercially available head immobilization devices: the Ossur Philadelphia Tracheotomy Collar (two-part light, rigid, polyurethane foam with Velcro fasteners; all plastic) plus the Aquaplast Thermoplastic mesh radiology mask, which was custom-fitted for each participant.

### Data analysis

All data were preprocessed and statistically analyzed using FMRIB's (Center for Functional Magnetic Resonance Imaging of the Brain, University of Oxford, UK) Software Library (FSL) version 6.00 (which utilizes MNI_152 coordinate space). Lower-level fMRI data processing was carried out using FMRI Expert Analysis Tool (FEAT). Preprocessing at the individual level included manual removal of skull and non-brain tissue from the anatomical and functional images. MCFLIRT (Jenkinson, Bannister, Brady, & Smith, 2002) motion correction was performed with extended motion parameters added to the model. The average mean motion displacement movement for these data was absolute=0.4 mm and relative=0.1 mm. All data were spatially smoothed using a 5-mm full-width at half-maximum Gaussian kernel. Registration of the functional images to the high-resolution anatomical images was performed outside of the FEAT, using FLIRT (FMRIB's Linear Image Registration Tool), selecting the options: mutual information cost function and sinc interpolation (Blackman, width of sinc window=7 voxels). Each participant's first-level FEAT registration file was updated with the FLIRT registration conducted outside of FEAT prior to the higher-level analyses.

Explanatory variables (EVs) were created at the first levels for nipple imagine model (NIM), NIS, clitoris imagine model (CIM), CIS, nipple touch model (NTM), NTS, clitoris touch model (CTM), CTS, imagine speculum (IS), and imagine dildo (ID). The first-level basic contrasts were set up for all EVs >0 and <0 (0=global baseline). Differential contrasts were also set up to compare each ‘stimulation’ condition (stimulate) with its ‘control’ condition (model): NIS>NIM, CIS>CIM, NTS>NTM, and CTS>CTM. Additional differential contrasts comparing across imagined stimulation and physical stimulation conditions were also set up: NIS>NTS; NTS>NIS; CIS>CTS; CTS>CIS. Contrasts were also set up to compare the two additional imagery conditions, ID>IS and IS>ID.

First-level analyses were conducted with a high-pass filter cutoff set at 180 s. FILM (FMRIB's improved linear model) prewhitening option was selected to improve estimation efficiency. The data were convolved using a double-gamma Hemodynamic Response Function (HRF) without temporal derivatives. The EVs were used as regressors to determine the average activity elicited by each condition. The data at first levels were corrected for multiple comparisons using a cluster-forming threshold of *z*=1.65 and a cluster-significance threshold of *p*<0.05. The output files (contrast of parameter estimates or ‘cope’ files) were then used in the higher-level analysis to determine mean group effects and to perform contrast analyses between the conditions.

Higher-level analyses were performed using FMRIB's local analysis of mixed effects (FLAME 1), which employs Metropolis-Hastings Markov chain Monte Carlo sampling to correct for multiple comparisons. To explore the data, a whole brain group analysis was conducted using a cluster-forming threshold of *z*=1.65 and a cluster-significance threshold of *p*<0.05. As the activity of the imagined stimulation differential contrasts (CIS>CIM; NIS>NIM) was significantly and unexpectedly greater than the activity observed in the physical stimulation differential contrasts (NTS>NTM; CTS>CTM), it was determined that the results of the differential contrasts for the subsequent group analyses for this data set should be contrast-masked post-threshold with the constituent basic contrast conditions greater than baseline to assure that the activity observed in the differential contrasts was positively driven. For example, the differential contrast CIS>CIM was contrast-masked with the positive voxels of each of the basic contrasts, specifically CIS>0 and CIM>0 (greater than global baseline) assuring that the results of all differential contrasts reflect only activity above the global baseline. This was done for all differential contrasts.

All higher-level analyses of this data set were corrected for multiple comparisons and contrast-masked post-threshold with the voxels above baseline as described. For the contrasts involving the physical and imagined stimulation of the nipple and clitoris, the cluster-forming z was set at 1.0, cluster-significance threshold *p*=0.01. For the contrasts comparing the imagery of the dildo (ID) and the speculum (IS), the cluster-forming threshold was set at *z*=1.65, *p*=0.05.

## Results

### Physical versus imagined stimulation of the clitoris and nipple

**Fig. 1:**
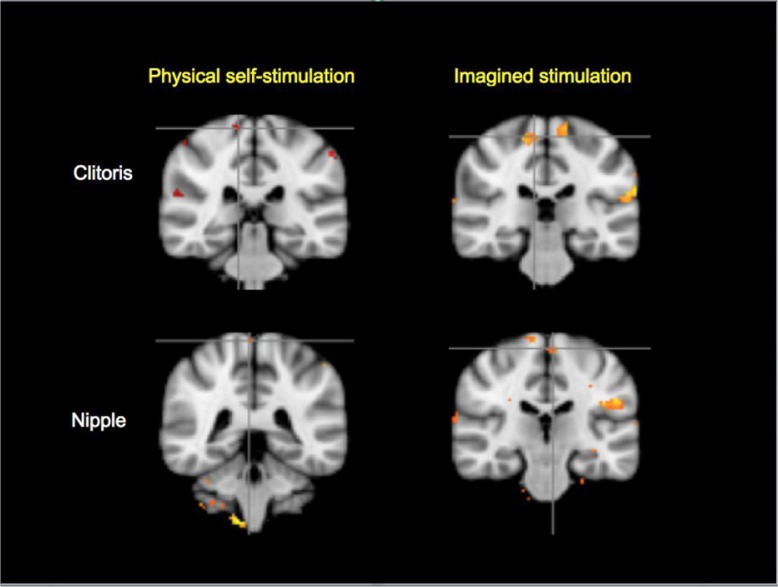
Paracentral lobule ‘genital sensory’ cortical activations in physical self-stimulation and imagined tactile stimulation of the clitoris and nipple, indicated by location of the crosshairs (cluster *z*=1.0, *p*<0.01, *N*=11). In this and subsequent figures, the following convention was used: the MNI_152 slice coordinates, (*y*=coronal, *z*=axial) are specified clockwise, starting from upper left. In this figure, *y*=−32/−28/−24/−40. The contrasts shown above, and in Figs. 2 and 3, clockwise from upper left are CTS>CTM, CIS>CIM, NIS>NIM, and NTS>NTM, respectively. Abbreviations and analysis are specified in Methods section.

The paracentral lobule (genital sensory cortex) was activated by both physical and imagined stimulation of the clitoris and nipple (Fig. 1). There were no significant differences between physical and imagined stimulation in that region for these conditions.

**Fig. 2:**
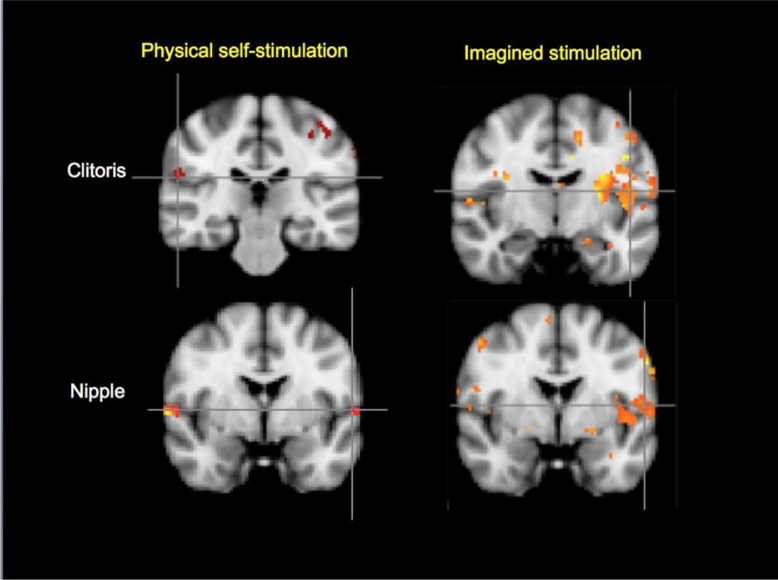
Secondary somatosensory cortical activations in physical and imagined stimulation of the clitoris and nipple, (cluster *z*=1.0, *p*<0.01, *N*=11). Top left: clitoris, physical stimulation (OP1 right side of brain); top right: clitoris, imagined stimulation (OP4 left). Bottom left: nipple, physical stimulation (OP4 right); bottom right: nipple, imagined stimulation (OP4 left). OP1, parietal operculum 1; OP4, parietal operculum 4; MNI_152 coordinates: *y*=−26/−10/−2−2.

As shown in Fig. 2, activation of the left parietal operculum (OP4) (i.e. left secondary somatosensory cortex) was observed for imagined stimulation of the clitoris and nipple, while physical self-stimulation of the clitoris and nipple activated right parietal operculum (OP1 and OP4, respectively).

**Fig. 3:**
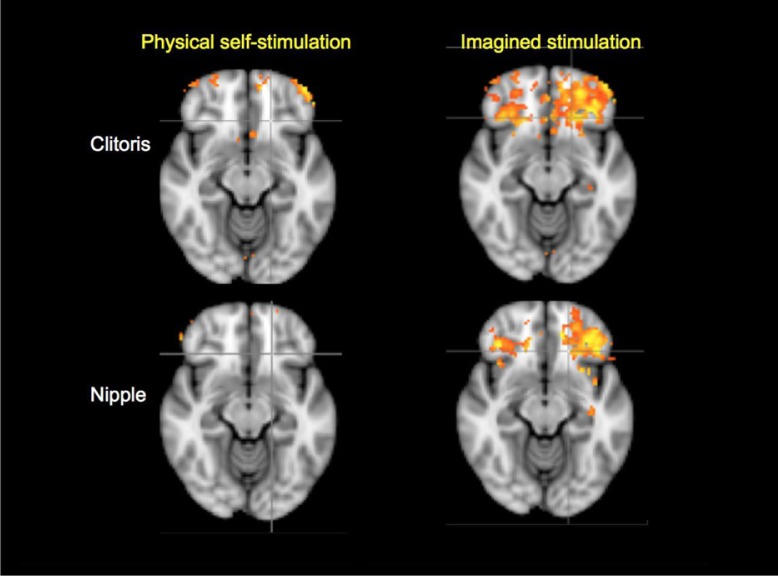
Frontal cortical activations in physical and imagined self-stimulation of the clitoris and nipple (cluster *z*=1.0, *p*<0.01, *N*=11; MNI_152 coordinate: *z*=−12/−12/−12/−12).

The imagery conditions resulted in greater observed activation of the frontal pole and orbital frontal cortex than did the physical stimulation conditions (Fig. 3).

**Fig. 4:**
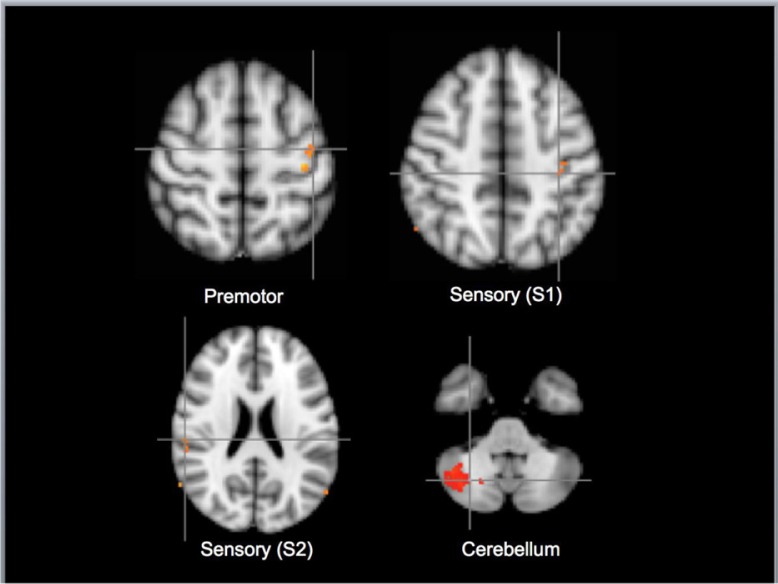
Regions activated by tactile self-stimulation of the clitoris>imagined self-stimulation of the clitoris. Top left: premotor cortex BA6 left side of brain. Top right: primary somatosensory cortex BA3a left. Bottom left: secondary somatosensory cortex OP1 right. Bottom right: cerebellum right. (Cluster *z*=1.0, *p*<0.01, *N*=11; MNI_152 coordinate: z=56/48/−42/22). The above contrast is CTS>CIS.

As shown in Fig. 4, a direct comparison of tactile self-stimulation of the clitoris with imagined tactile self-stimulation resulted in significantly greater activation of the cerebellum, primary somatosensory cortex (hand region), and premotor cortex.

Regions that were activated significantly only during the imagined, but not the tactile, self-stimulation conditions include the insular cortex, amygdala, hippocampus, and inferior parietal lobule (not shown).

### Imagined stimulation by dildo versus speculum

**Fig. 5:**
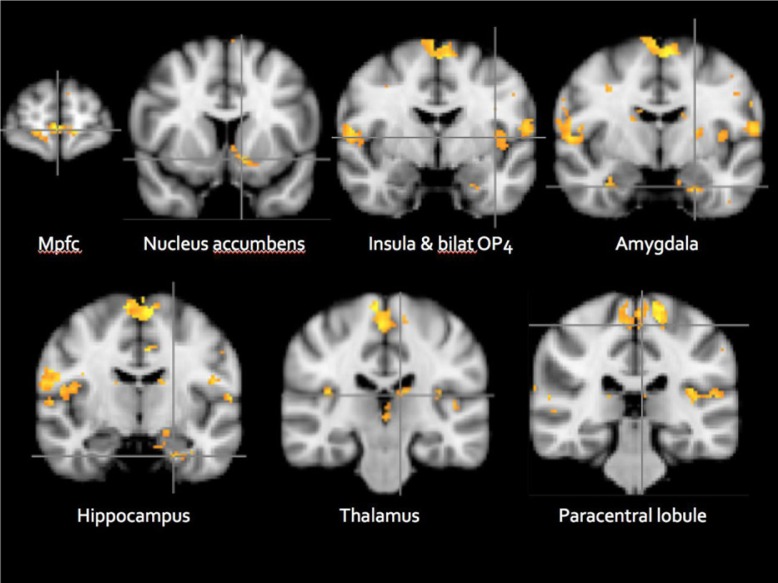
Imagined stimulation by dildo>speculum (ID>IS) (cluster *z*=1.65, *p*<0.01, *N*=11).Mpfc, medial prefrontal cortex; Bilat Op4, bilateral parietal operculum 4; MNI_152 coordinate: *y*=62/10/−4/−6/−30/−24/−10.

As shown in Fig. 5, the comparison: imagined dildo stimulation>imagined speculum stimulation revealed significant activation in the paracentral lobule and secondary somatosensory cortex (OP4), thalamus, frontal and insular cortices, amygdala, nucleus accumbens, and hippocampus. In addition, activations for this contrast were noted in the cerebellum and medulla (data not shown).

There were no significant results (no brain regional activations) for the comparison: imagined speculum stimulation>imagined dildo stimulation.

### Participants’ ratings of vividness of imagery and sexual arousal

**Fig. 6:**
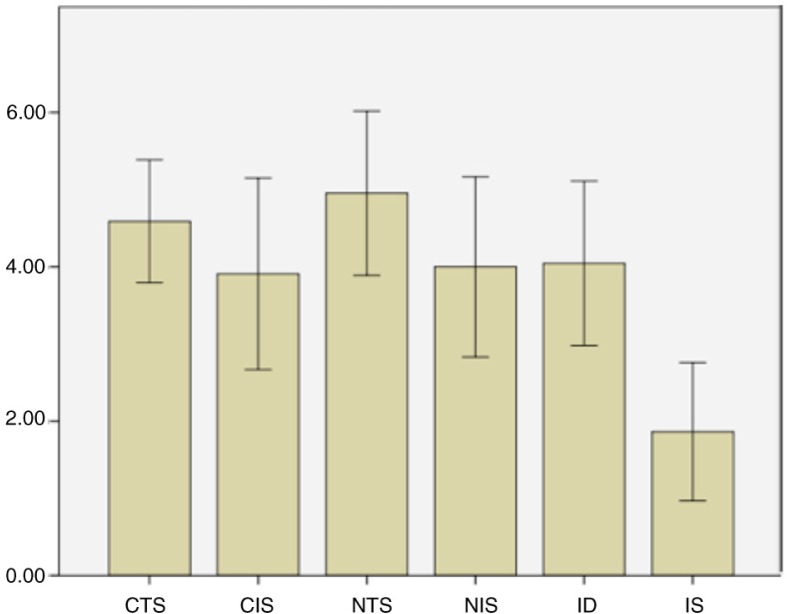
Participants’ reported mean levels of sexual arousal for tactile and imagined tactile stimulation on scale of 1 (no arousal) to 7 (high arousal). N=11; error bars ±2 SEM.CTS, clitoral touch stimulation; CIS, clitoral imagined stimulation; NTS, nipple touch stimulation; NIS, nipple imagined stimulation; ID, imagined dildo stimulation condition; IS, imagined speculum stimulation condition.

Participants reported comparably high levels of vividness across all imagery conditions. As seen in Fig. 6, the mean level of arousal produced by imagery versus tactile self-stimulation of the nipple or clitoris was comparable to the extent that there was no significant difference between them. The level of arousal produced by imagery of dildo stimulation was significantly greater than that produced by the imagery of speculum stimulation [*t*(8)=2.57, *p*=0.03].

## Discussion

The present findings are consistent with a growing body of evidence that 1) brain regional activity elicited by imagined stimulation of specific body parts overlaps with that elicited by actual tactile stimulation and 2) there are important similarities, as well as differences, in how the brain represents physical and imagined stimulation.

Noteworthy, and unexpectedly, imagining dildo stimulation generated extensive brain activation in regions previously shown to be active in the process of genital stimulation leading up to and including orgasm (Komisaruk et al., 2004; Komisaruk & Whipple, 2005; Komisaruk, Wise, Frangos, & Allen, 2010; Komisaruk, Wise, Frangos, Birbano, & Allen, 2011; Wise, 2014; Wise et al., 2012; Wise, Frangos, & Komisaruk, (Submitted for publication)), whereas imagining speculum stimulation generated virtually no brain activation. Just thinking about stimulation by a dildo resulted in bilateral activation of the genital sensory cortex (paracentral lobule), as well as the secondary somatosensory cortex (the parietal operculum, OP4). The OP4 region was shown previously to have somatotopically organized body maps for hands (Eickhoff, Schleicher, Zilles, & Amunts, 2006), feet (Young et al., 2004), penis (Kell, von Kriegstein, Rösler, Kleinschmidt, & Laufs, 2005), and anus (Eickhoff, Lotze, et al., 2006). In addition to these primary and secondary somatosensory cortical activations, the left thalamus, left insula, left hippocampus, left nucleus accumbens, bilateral amygdala, cerebellum, medulla, and the medial frontal cortex were activated in the imagine dildo stimulation>imagine speculum stimulation comparison. Consistent with the present findings of an involvement of the left insula, Ortigue, Grafton, and Bianchi-Dimichell (2007) and Bianchi-Demichell and Ortigue (2007) reported the activation of the left anterior insula in relation to the memory of sexual stimulation. We concur with their interpretation that ‘… the left anterior insula plays a pivotal role in autonomic, emotional, and reward processes….’ (Bianchi-Demichell & Ortigue, 2009, p. 549).

There was no significant regional activation for the comparison: imagine speculum stimulation>imagine dildo stimulation. The dildo imagery was rated as significantly more sexually arousing than speculum imagery, which suggests that the degree of sexual arousal may be more salient than the vividness of imagery in terms of how brain activity, measured by fMRI, represents the imagined stimulation. Furthermore, as no participant rated any of the imagery conditions as aversive, it is likely that the difference between the dildo imagined and speculum imagined stimulation conditions is not a result of the speculum stimulation imagery being aversive.

Based on recent evidence reported in the literature regarding tactile imagery (Olivetti Belardinelli et al., 2009; Yoo et al., 2003), we expected that the tactile stimulation conditions would result in greater activation of the somatosensory cortices than the imagery conditions. Contrary to this prediction, greater than expected activity was observed in the genital sensory cortex (paracentral lobule of S1) in the imagery conditions, whereas less robust activity than expected was observed in the tactile stimulation conditions. We believe that a number of factors may have contributed to the lack of a robust response to the tactile stimulation conditions. In terms of the study design, the protocol sequence consistently began with the imagery trials to avoid the potential priming effects that actual tactile stimulation could induce. Thus, the conditions were not counterbalanced, and as a result, there may have been a habituation/order effect contributing to the lack of robust response to the tactile stimulation trials.

Another contributing factor might be due, at least in part, to the characteristics of the study's participants. All participants described themselves as being ‘consistently highly orgasmic’. There is support in the literature for correlations among orgasm reliability, hypnotic suggestibility, and imagery ability (Bridges, Critelli, & Loos, 1985; Harris, Yulis, & Lacoste, 1980). Consequently, the high degree of suggestibility of these participants may have biased the results toward more robust imagery activation than expected.

The absence of a robust brain response to the tactile stimulation conditions may have also been due to the explicit modeling control being too similar to the actual tactile stimulation, which would minimize their differences, thereby appearing as an absence of activation. To address this, we reduced the cluster-forming threshold in order to discern differences between the stimulation and modeling conditions. This, in addition to the small sample size, could have weakened the conclusions that can be drawn from that part of the study. In addition, it could have compromised the distinction between the tactile and imagery ‘maps’ for the clitoris and nipple within the primary and secondary somatosensory cortices.

The imagine dildo stimulation>imagine speculum stimulation contrast provides support for the capacity of imagery to activate brain regions implicated in the processing of bodily sensation, sexual stimulation, reward, and orgasm. This process may underlie the ability of some women to induce orgasm by imagery alone, in the absence of physical stimulation (Whipple, Ogden, & Komisaruk, 1992). The dildo (erotic) versus speculum (vivid but non-erotic) stimulation imagery findings provide evidence that activity in the following brain regions correlates with ‘erogenous’ experience: hippocampus, amygdala, insula, accumbens, medial prefrontal cortex, and primary and secondary sensory cortices. Overall, the results are consistent with those of our previous study (Komisaruk, Wise, Frangos, Liu, et al., 2011), localizing the sensory representation of the physical stimulation of the nipple and clitoris to the genital sensory cortex (paracentral lobule), and now extending this finding to include the representation of imagined stimulation of these body parts.

Identifying the somatosensory maps for physical stimulation of the female body could be useful in the development of effective treatments for disorders that predominantly or exclusively affect women, such as persistent genital arousal disorder (Leiblum & Nathan, 2001), pelvic pain conditions, vulvodynia (Di Noto, Newman, Wall, & Einstein, 2012), dyspareunia, anorgasmia, and hypoactive sexual desire disorder.

Two additional questions are raised by these findings. Is the ability to activate brain regions involved in bodily sensation, sexual stimulation, reward, and orgasm by imagery alone restricted to individuals with high levels of hypnotic suggestibility or vividness of imagery? Is the preponderance of left-sided brain activation (amygdala, insula, hippocampus, and frontal cortex) observed in the ‘sexually arousing’ imagery conditions related to reports that increase in left temporal and frontal regional activation is associated with enhanced responsivity to rewarding and positive stimuli (Davidson, 1992; Tomarken & Keener, 1998)?

Evidence that frontal asymmetry is involved in emotional regulation (Allen, Harmon-Jones, & Cavender, 2001) has led to recent applications such as real-time functional magnetic resonance imaging (rtfMRI), in conjunction with EEG, as a therapeutic tool, using neurofeedback. The goal of this approach is to volitionally increase activity in the left amygdala (Zotev, Phillips, Yuan, Misaki, & Bodurka, 2014) and insula (Veit et al., 2012), regions that are associated with enhanced mood regulation and reduced symptoms of anxiety and depression. Perhaps, pleasurable tactile imagery could be a naturalistic way of enhancing mood states, as suggested by yogic tradition. Real-time fMRI may be a means of providing insights into potential therapeutic applications of imagery.

## Conclusion

The present findings provide evidence that mental imagery activates brain regions implicated in bodily sensation, orgasm, and reward that overlap with, and differ from, the brain regions that respond to tactile self-stimulation. Furthermore, the hippocampus, amygdala, insula, nucleus accumbens, medial prefrontal cortex, and primary and secondary sensory cortices may participate in ‘erogenous’ experience. The process by which some individuals are able to generate orgasm by imagery in the absence of physical stimulation (Whipple et al., 1992) may be mediated by their ability to volitionally activate these brain regions. It may be feasible to use fMRI neurofeedback training to facilitate activation of these regions therapeutically.
